# Empowering Advocacy: A Theatre Pedagogy Training With Occupational Therapists

**DOI:** 10.1155/oti/3035183

**Published:** 2025-09-09

**Authors:** Panagiotis Barmpagiannis, Olga Chazapi

**Affiliations:** ^1^Occupational Therapy Department, University of Western Macedonia, Kozani, Greece; ^2^Department of Secondary Education, Greek Ministry of Education Religious Affairs and Sports, Marousi, Greece

## Abstract

**Introduction:** Advocacy is a cornerstone of occupational therapy practice, yet systemic barriers and complex interpersonal dynamics often hinder its effective implementation. This study was aimed at exploring the essential elements that occupational therapists perceive as essential for advocating effectively on behalf of the individuals they collaborate with.

**Methods:** Eleven occupational therapists participated in a 3-month educational program grounded in theatre pedagogy, an experiential learning approach designed to foster self-reflection and practical skill application. Following the program, semistructured interviews were conducted, and the data were analyzed using thematic analysis to identify key elements of advocacy.

**Findings:** The analysis revealed three core themes: occupational justice awareness, creativity and flexibility, and perspective expansion. Participants highlighted the importance of recognizing systemic inequities and promoting inclusive practices to achieve occupational justice. Creativity and adaptability were identified as essential for tailoring advocacy strategies to diverse contexts and challenges. Perspective expansion, encompassing cultural humility and reflective practice, emerged as vital for understanding the multifaceted dynamics of advocacy and the evolving roles of therapists in addressing personal needs.

**Conclusion:** This study contributes to the growing body of knowledge on advocacy in occupational therapy by identifying specific competencies that enhance advocacy efforts. The findings underscore the need for targeted training programs and resources that integrate experiential learning methodologies to strengthen these competencies. By cultivating these skills, occupational therapists can become more effective agents of change, advancing relationship-focused care and societal equity.

## 1. Introduction

Occupational therapy is a relationship-focused profession [1] that promotes health, well-being, and quality of life across the lifespan, delivered with critical consciousness and cultural humility [2, 3]. In this role, occupational therapists (OTs) act as key advocates, working to ensure inclusivity, accessibility, and equity for the individuals [4, 5]. However, the complexity of systemic barriers, policies, and cultural contexts often challenges OTs' advocacy efforts [6–8]. This calls for expanding the professional capacity of OTs in decision-making processes and cultivating resilience when facing these challenges, which is essential to their professional identity [9, 10].

Advocacy remains an essential dimension of occupational therapy, yet it is inconsistently addressed in both education and practice [11]. Among the core competencies identified in the literature are policy literacy, collaboration with stakeholders, community empowerment, outcome communication, and active engagement with professional bodies [12]. These elements span across micro, meso, and macro levels of intervention and are closely linked to occupational justice and systemic change [13]. Despite its recognized importance, many therapists report limited opportunities for structured development of these skills following their entry-level education.

In recent years, several international and organizational efforts have been aimed at strengthening advocacy capacity within the profession. The World Federation of Occupational Therapists (WFOT) has actively supported initiatives promoting advocacy, such as the successful legislation campaign for occupational therapy in Argentina [14], policy revisions that established OT as a statutory service in Norway [15], and the enrichment of Arabic-language OT content to support access and inclusion in the Arab world [16]. These contemporary examples underscore the global momentum around advocacy in occupational therapy but also reveal a gap in understanding how such movements translate into structured educational experiences and sustained professional competencies.

It has been argued that one effective way to enhance professional capacity is through experiential learning, which immerses participants in real-life scenarios, encouraging active engagement, reflection, and skill application [16]. This hands-on method deepens understanding by allowing professionals to navigate challenging situations [17], defining it as valuable for developing advocacy skills in occupational therapy. By integrating experiential learning into their training, OTs can better adapt to the systemic challenges they face and strengthen their roles as advocates for people's needs and wishes [18].

Theatre pedagogy, traditionally used in teachers training programs, is an experiential learning approach that offers an immersive way of addressing professional challenges [19]. As a creative educational tool, it is claimed to transform theoretical knowledge into lived experiences, enabling practitioners to engage with complex concepts like advocacy through embodied learning [20]. This same approach could possibly be considered equally valuable for OTs, who, according to Beers et al. [21], must navigate complex interpersonal and systemic dynamics in their roles as advocates.

Through active participation, reflection, and the dismantling of preconceptions, theatre pedagogy training enables practitioners to explore advocacy from both a personal and professional lens [22]. By engaging in such creative and reflective processes, OTs are empowered to re-examine their roles and consider new strategies for supporting their people's needs within complicated systems [23, 24]. Educationally, it has been used in other related professional disciplines such as medical education [25], nursing training [26], engineering and psychology [27], and for Greek professors in higher education [28].

In light of these considerations, the aim of this study was to investigate OTs' perspectives on the key elements they consider essential for effectively advocating on behalf of the individuals they collaborate with. Given the challenges OTs face in navigating systemic and interpersonal complexities, theatre pedagogy was selected as the educational tool for this research due to its capacity to foster experiential learning and self-reflection.

## 2. Research Methodology

### 2.1. Participants

The people in the sample volunteered to participate in the research, which was announced in various social media (Facebook and Instagram, registered trademarks of Meta Platforms Inc.) through an independent organization, named “Spot – An Inclusive Space for Education and Therapy.” Participants were required to be professional OTs and work in special therapy units, which offer individual occupational therapy services within a multidisciplinary setting of speech therapy, psychology, and special education. This selection was made to ensure a clear, established working context, reflecting the focus of the services provided. In these units, the majority of users of these services are children or adolescents with their families.

Due to the experiential and interactive nature of theatre pedagogy, participation was limited to a small number of professionals. In this study, the sample comprised 11 OTs. Priority for inclusion was given to individuals who responded first to the invitation. All data retrieved are shown in Table 1.

### 2.2. Data Collection Process

Before the implementation of the training program, a consent form and a demographic scale was constructed by the authors and completed by the participants. The purpose of the demographic scale was to collect data on age, gender, years of experience as practicing OTs, working hours per week, and asked if they knew the theoretical and/or have ever used, in any case, any practical aspect of advocacy in occupational therapy. Based on Dhillon et al. [29], the theoretical and practical aspect of advocacy was presented in writing to participants as follows: “theoretical refers to understanding advocacy concepts and ethical principles guiding OTs” and “practical involves real-world application, such as advocating for clients' needs, ensuring service access, and addressing systemic obstacles to influence policy and community practices.”

After the implementation of the training program, two semistructured interview questions were designed according to the main purpose of the study and the thematic analysis theory [30]. The main research question was “*what do you believe are the most important skills or knowledge areas that occupational therapists need to effectively advocate for their clients?*” and the catch-all question was “*is there anything else you would like to add regarding your perspectives and experience during the program on advocacy for clients?*”

This primary research question was designed to explore whether participants, drawing from both their personal and professional experiences and their engagement in the training program, could identify the most critical skills or knowledge areas required for effective advocacy on behalf of the individuals they work with. Rather than focusing on a summary of the program content, the question was aimed at eliciting thoughtful reflections on whether the program, in combination with their existing experiences, provided clarity or insight into these key advocacy competencies.

### 2.3. Coding and Analysis

The study followed Braun and Clarke's [31] six-phase framework for doing a thematic analysis. The study followed a qualitative descriptive design with a constructionist orientation, which acknowledges that meaning is coconstructed through language, experience, and context. This approach was chosen for its flexibility and its capacity to highlight patterns across participants' narratives, while respecting the relational and situated nature of qualitative knowledge. The authors first became familiar with the data through active rereading and then generated and discussed initial codes. Afterwards, each author categorized codes into potential meaningful groups, trying to identify relationships and patterns. In this manner, subthemes emerged, and then, a coherent set of themes was produced collaboratively. The analysis was based on the audio-recorded data collected and transcribed from the interviews. The researchers advocate that there could be multiple and different interpretations [30].

Both authors had received prior training in qualitative research methods and had previous experience with thematic analysis in similar contexts. This background contributed to the credibility and consistency of the analytic process. Reflexivity was an ongoing process throughout the study. As both authors were involved in the facilitation of the training and the subsequent analysis of the interviews, regular discussions were held to acknowledge positionality, mitigate potential biases, and reflect critically on the power dynamics inherent in their dual roles. These reflections were recorded in field notes and integrated into the analytic dialogue to ensure transparency and accountability in interpretation.

For the purpose of this paper, all data was translated in English, trying to remain faithful to the original transcripts, despite some proverbs or Greek expressions that could not be accurately rendered in order to make sense.

### 2.4. Process and Educational Program

This research began in October 2023, and the participants engaged in a 3-month educational program consisting of six sessions (two per month, 3 h each). The structure of each session was grounded in the theatre pedagogy method developed in Greece [32–34], which emphasizes experiential learning through a four-phase structure aiming at progressive engagement with the theme, role exploration, presentation of group-created scenes, and analysis of the experiential process. While this method offers a consistent scaffolding for experiential engagement, the content, interactions, and outcomes within each session are inherently dynamic and emergent. Rather than prescribing specific learning objectives, the method is aimed at creating the conditions for participants to explore, discover, and construct meaning through embodied experience and group process [35, 36].

The topics of the six sessions were drawn from the Everyday Advocacy Decision Guide of the American Occupational Therapy Association [36] and are presented in Table 2. In addition to the shared four-phase structure, each session was also built around a central theatre technique [30–36] and utilized specific materials that supported embodied and symbolic exploration of the theme. These elements are included in the third and fourth columns of Table 2 to enhance clarity and reproducibility. Each session was thematically distinct but followed the same underlying structure. All participants attended all six sessions of the training program. The program was cofacilitated by both authors.

The first author has completed a 3-year formal training in theatre pedagogy and holds a master's degree in adult education with a focus on art-based learning. The second author has also received a 3-year training in theatre pedagogy. Both authors have previously facilitated theatre pedagogy–based workshops in professional and educational contexts, drawing on their training to design experiential learning environments. Their experience includes the implementation of similar programs in multidisciplinary therapy teams and adult education settings. Additionally, the first author integrates theatre pedagogy in undergraduate occupational therapy education and has disseminated related work regarding its pedagogical application within academic contexts [37].

The first phase (activation) was aimed at fostering engagement through movement or symbolic activities that introduced the session's theme in a nonverbal and embodied way. Depending on the focus, participants explored abstract ideas through playful interaction, sensory awareness, and body-based exercises that encouraged presence and spontaneity. One facilitator provided detailed verbal instructions to guide the activity, while the other actively participated alongside the group to model involvement and cultivate group cohesion. As the sessions progressed, instructions became increasingly minimal, allowing space for participants' own interpretations and expression to emerge more organically.

The second phase (deconstruction) used improvisation, image theatre, and role-play to explore relational dynamics relevant to the advocacy topic. Participants embodied roles that revealed tensions around power, responsibility, inclusion, or silence, depending on the theme of the session. These interactions invited internalized assumptions, professional habits, and social norms. The facilitators assigned roles, provided initial framing, and intervened selectively to sustain emotional safety and depth of engagement. As in the first phase, facilitation became more restrained over time, encouraging participant-led meaning making.

In the third phase (reconstruction), participants worked in small groups to cocreate symbolic representations of alternative advocacy responses. These ranged from theatrical scenes and frozen images to collective actions and gestures. The goal was to imagine and embody new relational possibilities grounded in creativity, solidarity, and professional insight. The facilitators adopted a supportive, nondirective role—available to clarify, encourage, or offer prompts as needed but deliberately stepping back to center participant autonomy in the creative process.

The final phase (reflection) involved discussion, connecting experiential learning with theoretical constructs and clinical realities. Participants articulated emotions, realizations, and dilemmas that emerged during the session and discussed how these might influence their everyday practices. The conversation was guided primarily by the participants themselves, with facilitators offering only general, open-ended questions intended to highlight key thematic links and foster the integration of embodied insight with concepts such as occupational justice, identity, and equity. This phase emphasized dialogue, ownership of meaning, and cultural humility in interpretation.

As both authors also facilitated the training sessions, reflexivity was an ongoing process throughout the study. Regular discussions were held to acknowledge their dual roles and to reflect critically on how their positionality and presence may have shaped the group dynamics and the analytic interpretations. These reflections were recorded in field notes and informed the collaborative data analysis process, contributing to the study's transparency and trustworthiness. Reflexivity was also approached as an ethical commitment: the authors actively sought to minimize power hierarchies during facilitation, maintained reflective journals to monitor their assumptions and emotional responses, and intentionally questioned their interpretive authority during analysis to protect participant perspectives.

To further support transparency and potential replication, detailed written records of the session designs and facilitator plans for each meeting—including specific activity descriptions and sequencing—are available upon request for readers or practitioners interested in adapting the method in their own settings.

The consent form and the demographic scale were completed at the beginning of the study, as opposed to the interviews that were conducted after the implementation of the program, between January and February 2024, face to face.

### 2.5. Ethical Consideration

All participants were fully informed about the purpose of the study, the use of its results, and their rights as participants through a detailed consent form, which they reviewed and signed before participating. In alignment with the General Data Protection Regulation (GDPR), all data were collected, stored, and analyzed with strict adherence to confidentiality measures. The research was approved by the Research Ethics Committee of the University of Western Macedonia, Greece. Participants received no financial or material compensation for their involvement in the study.

## 3. Results

Three main themes emerged and can be identified along with the subthemes in Table 3. This section includes excerpts from the original transcript, in an attempt to present the personal experiences and thoughts of the participants themselves.

### 3.1. Occupational Justice Awareness in Everyday Life

This first theme highlights participants' awareness of occupational justice in daily life, both as OTs and community members, and their efforts to remain mindful of its manifestations in everyday actions. Their words touched on how systemic conditions, relational dynamics, and community contexts can either restrict or support individuals' ability to participate in occupations that are meaningful, chosen, and culturally valid.

#### 3.1.1. Equity Awareness

The differentiation between equity and equality led participants to reflect on their unseen personal privileges and barriers; sometimes, it is almost impossible to be aware of them. Participant 5 (P-5) exemplifies this by connecting theatre pedagogy to the power of privileges and its social impact.

…Through the “with-in-there” (theatre pedagogy technique), I experienced the huge difference between equity and equality. I won't forget the feeling after I left, because I was thinking that we can have equity, but…but I had never related my privilege to how it might affect someone else… or a family…or…or, I don't know… a whole community. (P-5)

Moreover, there were references about the lack of knowledge related to the practical implements of equity, and participants gave specific examples they had thought of during the program, such as how a fine is issued or how a state benefit is granted. In these reflections, equity was not only understood as a principle of fairness, but also as a condition that shapes who can access, afford, or sustain participation in daily occupations.

…It's like the fines they give… there, it's not e.g. 400 euros for everyone if you park illegally but a certain amount of the amount… of the payment… what's it called… of your annual income, e.g. 3 percent of your income, 3 percent of someone else's and so on…. (P-3)

#### 3.1.2. Relationship Between Client and Self

The therapist–client relationship emerged as a recurring theme throughout the interviews, with discussions focusing on the power dynamics in clinical settings, the relationship as a dynamic coeducational process, the mutual empowerment of both parties, and the necessity of a support system to enhance the therapeutic process and its participants.

…It's about being available for the client and walking together, co-training each other at the same time. This (process) invents how he does something and I invent how that was made possible… how it could be rendered in other ways… to whom else I can apply it to. It's a two-way alliance that one generously offers to the other. But it's not charity. (P-7)

Here, occupational justice was implicitly linked to the quality of the relationship, where participation was shaped through reciprocity, contextual sensitivity, and shared decision-making.

#### 3.1.3. Community Engagement

Issues regarding the power of advocacy and how its influence is constantly present, were raised by the participants. “…We must all be in this, together. Advocacy is walking down the street wearing something someone else wouldn't wear... either because they don't have the privilege, the power, they're ashamed, they won't let them? Everyone advocates, even at home, in the parking lot, at work, on the street. If I see you being beaten on the street, do I speak up or keep walking? …” P-8 says.

Participants stressed the need for active community involvement in addressing barriers to meaningful occupations, citing examples from recent Greek events, including the public murder of a queer person and the death of a motorcyclist outside parliament. In this subtheme, occupational justice was situated in public and relational spaces, where access to civic, expressive, and relational occupations depends on cultural visibility, safety, and collective voice.

…It starts with education, in school, in the streets, in your relationships... it is a matter of everyone's concern… we must, we have to say it *ο*ut loud... for everyone to hear. (P-4)

### 3.2. Creativity and Flexibility

Creativity was captured as thinking outside the conventional methods to develop innovative strategies, and flexibility was captured as the ability to adapt the OTs' approaches based on the evolving needs and requests.

#### 3.2.1. Seeking Partners

“…It's hard to be lonely at work…”, P-1 stated. Healthy relationships with colleagues, person's families, and partners are cited as crucial to OT practice. Advocacy creates a safer space for individuals, but therapists must also feel secure in their own work environment to achieve this.

...I remember at the previous unit I worked in, the parents were ultimately the most supportive of me advocating in their child's school environment. Neither the boss, nor the co-workers… no one, I'm telling you…. (P-9)

Most participants cited various reasons for seeking individuals or organizations with more clinical experience or specialized advocacy knowledge, emphasizing the collaborative nature of these efforts. P-3 said “…Early on, I read about advocacy, but I had never put so much emphasis on the collaborative nature of the issue….”

#### 3.2.2. Adaptive Techniques

In reviewing the range of the occupational therapy process, it was mentioned that OTs work by utilizing the people's strengths for active participation and engagement in everyday activities. Starting with “...with so many tools and so many techniques, you can use anything in the room...you look around and just choose what works best for you...it's all there if you think about how...” (P-11); parallels were drawn, such as “…but isn't that occupational therapy, working with what we have?... I advocate by any means, in any place and in the way that suits me best at the time…” (P-6).

Critical thinking and the ability to adapt the knowledge and the professional capacity were identified as useful components that would help the participants take action and advocate.

#### 3.2.3. Problem-Solving

Problem-solving was referred to as a critical element of occupational therapy for addressing and overcoming not only the diverse and complex challenges of the individuals but also the therapists too.

…What is referred as “the problem” needs a lot of thinking. Who it's a problem for, what it serves, who it serves, how. It's not that simple… especially if it has to do with you as a therapist…. (P-2)

Addressing and solving a problem involves personal factors of those involved. Participants noted that experience and theoretical knowledge provide multiple perspectives, enabling diverse thinking. “Feeling completely prepared and sure of your actions…” was recorded as a process that “…can never take place…” (P-10).

Advocacy, in my mind, is directly related to taking everyone's place, putting myself in everyone's shoes and finding an equal solution... co-constructing it with others? Even now that I say it, it sounds hard to find how to pursuit it.... (P-5)

#### 3.2.4. Collaborative Art

In the context of the experiential nature of the theatre pedagogy program, comments were made about the dynamics of a collective action. Mentioning the power of collaboration combined with theatre art, the OT as a political being was highlighted.

…the collaboration and what was happening every time...you know…everyone could contribute to the creation and be part of the process and the result. It wasn't mine or yours or anybody else's... together, united. It was like we marched peacefully…. (P-8)

Other narratives emerged, parallelizing creating art to being a lawyer, advocacy to a centipede that had to involve all the players in order to move, and even parallelizing finding strengths to meeting new people and connecting to them in a more intimate way.

…trying to advocate for someone is like a tandem bike, you know… with the four pedals…. (P-3)

### 3.3. Perspective Expansion

Critical thinking and redefining the familiar allow therapists to recognize factors affecting person engagement and their own biases in service delivery, fostering awareness of beliefs, cultures, and social or environmental dimensions for a more inclusive approach.

#### 3.3.1. Role Enrichment

The role of an OT is so complex and multifaceted because it includes so many, different, aspects. Advocacy, in particular, can be seen and identified even in simple actions and questions such as “…I hadn't thought that every time I ask whose problem is a child's stereotypical behavior, I'm advocating for the child and for neurodiversity…” (P-2).

Role enrichment insights emerged, highlighting both the potential and perceived shortcomings within occupational therapy, as professionals noted that long work hours hindered personal study and theoretical training.

…playing with so many roles (she means in the process of theatrical roles during the program), I thought that I have been taught too well to be a “therapist”…and…and in my everyday life I lack the time to think critically about what I want to change in my role, to see who I am… because I wonder how I could eventually be an advocate or when I have been an advocate but I haven't realized it…or…or…who I advocated for the most… I work so many hours…. (P-11)

#### 3.3.2. Communication Patterns

The language used in clinical settings focuses on weaknesses rather than strengths, and participants observed that emphasizing weaknesses reinforces them. P-7 said “…it's different to speak about autism and different to speak about someone's behaviors….” This idea emerged from OTs' increased awareness of their clinical practice during the educational program.

…I noticed that there are certain narratives and situations that we follow without wanting to, we haven't thought about them, it's...it's as if we are guided by a common agreement that was agreed upon many years ago and there is no room for doubt.... (P-6)

Awareness of communication patterns is a significant step for OTs, enabling shifts in their perspectives through language use “…we're used to communicate in ways that are somewhat specific, they don't change easily… they stay fixed... changing our words is a political act…” (P-10).

#### 3.3.3. Cultural Humility

OTs reported that they have learned to work based solely on their professional training, rather than understanding the person's worldview, actively listen, and learn from their experiences.

…thinking that we know, is the problem… that we know our children, our clients, what they mean when they tell us they are sleeping, they love... how am I supposed to know that when you say sleeping, you mean in a bed and not down on the floor? Or that you sleep at night? I think that the not knowing approach, involve a lot of aspects that I hadn't thought about and now suits me so much… because I now let the person across me direct me and I don't have the power of knowing.... (P-11)

Cultural humility emerged in different ways and narratives, suggesting a continuous involvement of OTs either in trainings or in supervisory meetings.

It still impresses me that even a small educational training to have, can open you up to tremendous ideas and thoughts… it's like learning how to learn constantly…. (P-4)

## 4. Discussion

The purpose of this study was to investigate OTs' perspectives on the key elements they consider essential for effectively advocating on behalf of the individuals they collaborate with. A thematic analysis was conducted, and three main themes emerged.

Participants mentioned that occupational justice awareness is needed in everyday life. They, mainly, focused on the variety of advocacy modalities and the differentiation between equity and equality, not only within the narrow boundaries of occupational therapy services but across society. Being political and acting as social change agents [38] is just the principle of extending public awareness to schools, streets, organizations, friends, and acquaintances, to public and private places. Simple acts of advocacy against inequalities make efforts more visible to OTs [6]. Increased involvement in advocacy facilitates its application.

The participants noted that equitable collaboration between person and therapist [4] changes the dynamics of the relationship, turning both parties involved into cotrainers and cotrainees, creating a circular causality that becomes critical of the authority of either of the two. As indicated in Pollard and Sakellariou (p. 648) [39]: “Knowledge is not always a means of domination and neither does possession of knowledge necessarily lead to power.” This allows participants to feel more relaxed, safe, and empowered to collaborate with individuals and their families.

This study, also, sheds light to the experiential learning and its benefits across the training of OTs, because professionals can actively get involved in a process that promotes critical thinking and self-reflection through personal experience and discussion [17]. Such attempts have been recorded in the past for other aspects of the occupational therapy practice [40, 41] and are inextricably linked to other scientific fields, such as to the roots of family therapy theory “…all doing is knowing, and all knowing is doing…” (p. 27) [42].

Through the participants' narratives, creativity arose as an integral part of experiential learning [16] that extends beyond the training of OTs. Creativity and flexibility, according to them, are key tools for strengthening the capacity for advocacy—tools that are frequently cited in literature [18, 19, 21]. Unhealthy relationships in the workplace [43] and lack of materials reduce flexibility and imagination but can also be driving forces for more creative and adaptive techniques.

Elements such as collaboration, team spirit, and problem-solving techniques were stated as crucial in creating an environment where professionals can share insights, support one another, and develop strategies in order to improve personal needs [22]. Fostering collaboration and teamwork-driven approaches allows OTs to address complex cases, enhance professional development, and improve advocacy skills by learning from each other's experiences [5]. Without coordination and unity among various stakeholders within the occupational therapy profession, advocacy efforts can become diluted and less impactful.

Participants' exploration of their roles and perspectives revealed another element useful for advocating on behalf of the people they work alongside. Through cultural humility and role enrichment, social–cultural prejudices can be overcome [3], and this way, practitioners become active listeners and increase their ability to seek, evaluate, and use information to promote advocacy. The frequent use of the term client across participant narratives reflects the enduring presence of this terminology in occupational therapy practice. Despite growing international emphasis on relationship-focused and inclusive language, such as “people” or “individuals,” the persistence of client highlights the embeddedness of traditional models in everyday professional discourse. This coexistence suggests an ongoing shift in how therapeutic relationships are conceptualized and articulated within the field. This can be achieved through recognizing and changing the way language is used, since speech as a form of communication has a political dimension [44] and can challenge sovereignty or social constructs such as able-bodiedness and disability.

Throughout the interviews, participants often described themselves as unfamiliar with specific strategies or structured approaches to advocacy, noting that such content had not been part of their previous education or training. The results support the findings of previous research, indicating the need for more training in clinical practice [18, 45]. Further research is needed on advocacy applications, considering Greek academic studies and participants' long work hours. High work demands and lack of time contribute to stress [46], potentially affecting the entire occupational therapy process.

## 5. Conclusion

This study explored the key elements that OTs consider essential for effectively advocating on behalf of the individuals they collaborate with. Through the thematic analysis of participants' reflections, three core themes emerged. Occupational justice awareness was highlighted as foundational, emphasizing the need for therapists to recognize and address systemic inequities that impact their people's access to meaningful occupations. Participants also identified creativity and flexibility as vital tools for adapting advocacy strategies to the unique contexts and needs of individuals, demonstrating the importance of innovative problem-solving in overcoming barriers. Finally, perspective expansion—including cultural humility, reflective practice, and enriched understanding of their professional roles—emerged as a critical competency for therapists striving to advocate with greater insight and effectiveness.

These findings underscore the importance of equipping OTs with the skills and knowledge to act as agents of change in both clinical and community settings. The identified elements provide a context for designing targeted training programs and resources that foster advocacy competence. Future research should further explore how these elements can be integrated into professional development to enhance the advocacy impact of OTs across diverse practice environments.

### 5.1. Limitations

There are some limitations to consider in this study. First, the recruitment process was conducted via social media, which may have restricted participation to OTs with internet access and those active during the recruitment period. Second, the sample was relatively small and homogeneous, consisting exclusively of OTs working in pediatric settings with children and adolescents. This may limit the transferability of the findings to other occupational therapy populations and practice areas, such as adult rehabilitation, mental health, or geriatric care.

Additionally, the dual role of the researchers as both facilitators and analysts may have introduced response bias, including social desirability or the Hawthorne effect, as participants may have been influenced by the perceived expectations of the study context. Although reflexive practices were employed, these dynamics cannot be fully eliminated.

Finally, the experiential nature of theatre pedagogy, while offering rich opportunities for critical engagement, may lead to variation in implementation depending on the cultural, institutional, or educational context. Adaptations to local frameworks and needs may be necessary. Further research is needed to explore how similar advocacy-focused training can be developed, contextualized, and evaluated across diverse occupational therapy settings.

## Figures and Tables

**Table 1:** Participant's data.

**Participant**	**Age**	**Gender**	**Years of experience as OT**	**Level of education**	**Working hours per week**	**Theoretical and practical aspects of advocacy**
1	25	Female	3	Bachelor	> 40	Only theoretical
2	43	Female	21	Bachelor	> 40	Neither
3	23	Female	1	Bachelor	> 40	Only theoretical
4	24	Nonbinary	2	Bachelor	> 40	Both
5	31	Female	9	Bachelor	> 40	Neither
6	35	Female	12	Bachelor	> 40	Neither
7	28	Male	5	Master processing	> 40	Both
8	29	Female	7	Bachelor	> 40	Neither
9	38	Female	16	Master	> 40	Only theoretical
10	31	Female	5	Bachelor	> 40	Neither
11	23	Female	1	Bachelor	> 40	Neither

**Table 2:** Topics, Theatre techniques, and materials of training program, according to AOTA's Everyday Advocacy Decision Guide.

**Items of Everyday Advocacy Decision Guide**	**Topics of the training program**	**Main theatre techniques**	**Main materials used**
Ways to advocate	Forms of advocacy: Individual, systemic, and legislative Collaboration in advocacy efforts Diverse populations	Image theatre and freeze frame	A4 sheets, post-it notes, markers

Establish credibility	Professional relationships OTs and stakeholders—Relation and communication Addressing bias to enhance credibility	Role-play and conscience alley	Chairs, name cards, simple props

Prepare for difficult conversations	Managing conflict Empathy and assertiveness Bureaucratic systems	Forum theatre	Everyday objects (e.g., bags and ropes)

Utilize strengths	Language of weaknesses and language of strengths Strength-based advocacy Leveraging strengths to overcome barriers	Mantle of the expert and hot seating	Printed profiles, role descriptors

Champion for OT	Promoting OT in multidisciplinary teams Highlighting OT's role in occupational justice	Teacher (therapist) in role and small group improvisation	Large paper, tape, space markers

Engage in active leadership	Leadership Inspire and guide Organizational policies	Improvisation and soundscapes	Fabric pieces, cups, paper objects

**Table 3:** Themes and subthemes.

**Themes**	**Subthemes**
Occupational justice awareness in everyday life	Equity awareness
	Relationship between client and self
	Community engagement

Creativity and flexibility	Seeking partners
	Adaptive techniques
	Problem-solving
	Collaborative art

Perspective expansion	Role enrichment
	Communication patterns
	Cultural humility

## Data Availability

The entire design, structure, content, and implementation of the training program are available from the first author.
